# Study of lipid profile and parieto-temporal lipid peroxidation in AlCl_3 _mediated neurotoxicity. modulatory effect of fenugreek seeds

**DOI:** 10.1186/1476-511X-11-16

**Published:** 2012-01-26

**Authors:** Yosra Belaïd-Nouira, Hayfa Bakhta, Mohamed Bouaziz, Imen Flehi-Slim, Zohra Haouas, Hassen Ben Cheikh

**Affiliations:** 1 Research unit of Genetic (02/UR/08-03), Laboratory of Histology and Cytogenetic, Faculty of Medicine, Monastir, Tunisia; 2 Laboratory of Bioprocesses, Center of Biotechnology, University of Sfax, Tunisia

## Abstract

**Background:**

Peroxidation of lipid (LPO) membrane and cholesterol metabolism have been involved in the physiopathology of many diseases of aging brain. Therefore, this prospective animal study was carried firstly to find out the correlation between LPO in posterior brain and plasmatic cholesterol along with lipoprotein levels after chronic intoxication by aluminium chloride (AlCl_3_). Chronic aluminum-induced neurotoxicity has been in fact related to enhanced brain lipid peroxidation together with hypercholesterolemia and hypertriglyceridemia, despite its controversial etiological role in neurodegenerative diseases. Secondly an evaluation of the effectiveness of fenugreek seeds in alleviating the engendered toxicity through these biochemical parameters was made.

**Results:**

Oral administration of AlCl_3 _to rats during 5 months (500 mg/kg bw i.g for one month then 1600 ppm via the drinking water) enhanced the levels of LPO in posterior brain, liver and plasma together with lactate dehydrogenase (LDH) activities, total cholesterol (TC), triglycerides (TG) and LDL-C (Low Density Lipoproteins) levels. All these parameters were decreased following fenugreek seeds supplementation either as fenugreek seed powder (FSP) or fenugreek seed extract (FSE). A notable significant correlation was observed between LPO_brain _and LDL-C on one hand and LDH_liver _on the other hand. This latter was found to correlate positively with TC, TG and LDL-C. Furthermore, high significant correlations were observed between LDH_brain _and TC, TG, LDL-C, LPO_brain _as well as LDH_liver_.

**Conclusion:**

Aluminium-induced LPO in brain could arise from alteration of lipid metabolism particularly altered lipoprotein metabolism rather than a direct effect of cholesterol oxidation. Fenugreek seeds could play an anti-peroxidative role in brain which may be attributed in part to its modulatory effect on plasmatic lipid metabolism.

## Background

A close relationship between lipid peroxidation and hypercholesterolemia and/or hyperlipidemia has been evidenced in plasma, liver and aorta through many studies in animals and humans [1]. These factors contribute to the process of several pathologies namely atherosclerosis, but also seem implicated in the physiopathology of neurodegenerative diseases particularly Alzheimer's disease (AD) [2]. Indeed, lipid peroxidation is considered as the most prominent form of oxidative damage in neurodegenerative lesions due to the brain's relative enrichment in polyunsaturated fatty acids [3]. On the other hand, an increasing number of reviews implicating cholesterol metabolism in the development of AD have been published [4] which suggest cholesterol as a target for treatment. However, although it is admitted that an animal model able to reproduce all the cognitive, behavioural, biochemical, and histopathological abnormalities observed in AD patients does not exist [5], a partial reproduction of some AD hallmarks, using Al salts, have been achieved [6].

In this study, we aimed firstly to evaluate the relationship between plasmatic cholesterol metabolism and lipid peroxidation in posterior brain (parieto-temporal and occipital regions), previously found to be preferentially affected by AD [7], using Al, a potential etiological factor in AD [8], as neurotoxicant. Chronic aluminum-induced neurotoxicity has been in fact related to enhanced brain lipid peroxidation [9] together with hypercholesterolemia and hypertriglyceridemia [10].

Moreover, in the search for a new drug which may offer neuroprotection by controling both hypercholesterolemia and lipid peroxidation, we have tested fenugreek seeds. Fenugreek (*Trigonella foenum-graecum)*, a legume cultivated predominantly in Asia, the Mediterranean and North African regions for the edible and medicinal values of its seeds, has been reported to have antioxidative and hypocholesterolemic properties [11]. *Trigonella *is also known for its multiple pharmacological effects including its antidiabetic, antineoplastic, anti-inflammatory, antiulcerogenic, antipyretic, antitumor and immunomodulatory effects [12]. The active components of fenugreek seeds behind their most common properties (i.e hypoglycemic, hypocholesterolemic, hypotriglyceridemic and antiperoxidative) have been described as polyphenolic flavonoids [13], steroid saponins [14], polysaccharides mainly galactomannans [15] and 4-hydroxyisoleucine [16]. Nevertheless, the neuroprotective effect of fenugreek seeds was mainly restricted to studies on diabetes [17-19] except for a single *in vitro *study which have demonstrated the acetylcholinesterase enzyme inhibitory potential of standardized extract of fenugreek seeds [12]. Thereby, the role of fenugreek seeds in neurodegenerative diseases and especially against aluminum-induced changes has not so far been considered.

## Results

### Lipid profile and glucose levels in blood

Plasmatic levels of total cholesterol (+42.3%), LDL-C (+63.52%), triglycerides (+81.81%), and glucose (+97.85%) were found to be significantly increased in Al-treated rats (Table 1), as compared to controls. No significant changes were observed in rats consuming fenugreek seeds and treated (AlCl3+FSP, AlCl3+FSE) or not (FSP, FSE) with AlCl3.

**Table 1:** Lipid profile and blood glucose level in different experimental groups.

Parameter	Experimental groups					
	**Control**	**AlCl_3_**	**AlCl_3_+FSP**	**AlCl_3_+FSE**	**FSP**	**FSE**

**TC**	1.56 ± 0.009	2.22a* ± 0.36	1.56^b* ^± 0.01	1.61^b* ^± 0.06	1.56 ± 0.08	1.55 ± 0.10
**HDL-C**	0.49 ± 0.02	0.44 ± 0.01	0.57^b*c ^± 0.01	0.50^bc ^± 0.004	0.61^a ^± 0.03	0.54^a ^± 0.01
**LDL-C**	0.85 ± 0.02	1.39^a*^ ± 0.03	0.89^b*^ ± 0.01	0.84^b* ^± 0.04	0.83 ± 0.05	0.80 ± 0.05
**TG**	0.66 ± 0.02	1.20^a* ^± 0.06	0.58^b* ^± 0.02	0.74^b*^ ± 0.04	0.58 ± 0.14	0.68 ± 0.03
**Glc**	8.39 ± 0.03	16.6^a*^ ± 1.30	8.18^b* ^± 0.08	7.85^b* ^± 0.29	8.31 ± 0.02	8.18 ± 0.24

Values are expressed as means ± SD; n = 10 for each treatment group.

^a ^Significant difference from the control group at p < 0.05.

^b ^Significant difference from the AlCl_3_-intoxicated group at p < 0.05.

^c ^Significant difference between AlCl_3_+FSP and AlCl_3_+FSE groups at p < 0.05.

* p < 0.001.

All parameters are expressed as mmol/l.

HDL-C level was unchanged in Al-treated groups (AlCl3, AlCl3+FSP and AlCl3+FSE), but significantly increased when fenugreek seeds were administrated alone either as FSP (+24.4%) or FSE (+10.2%).

### Estimation of lipid peroxidation levels (TBARS)

After 5 months of exposure to AlCl_3_, a significant raise of TBARS levels occurred increasingly in liver (+44.65%), plasma (+47.56%) and brain (+78.42%) whereas no significant changes were noted in (AlCl_3_+FSP) and (AlCl_3_+FSE) groups (Table 2). In (FSP) and (FSE) treated rats, TBARS levels were not significantly different from controls except in the liver where we noticed a slight but significant increase for both groups.

**Table 2:** Thiobarbituric acid-reactive substances (TBARS) and lactate dehydrogenase (LDH) activity in brain, liver and plasma of different experimental groups.

Parameter	Experimental groups					
	**Control**	**AlCl_3_**	**AlCl_3_+FSP**	**AlCl_3_+FSE**	**FSP**	**FSE**

**Brain**						
TBARS	3.43 ± 0.35	6.12^a* ^± 0.12	3.76^b* ^± 0.21	2.46^b* ^± 0.28	4.07 ± 0.02	4.15 ± 0.37
LDH	84.9 ± 1.27	115.8^a ^± 7.21	86.4^b ^± 3.65	93.3^a*b ^± 0.20	83.05 ± 1.58	83.13 ± 2.64
**Liver**						
TBARS	2.15 ± 0.18	3.11^a ^± 0.24	2.75 ± 0.20	2.32^b ^± 0.15	3.02^a ^± 0.07	2.88^a ^± 0.25
LDH	191.4 ± 6.45	282.2^a*^ ± 11.2	199.9^b*c*^ ± 0.80	185.5^b*c*^ ± 2.62	195.7 ± 3.49	184.9 ± 0.30
**Plasma**						
TBARS	0.82 ± 0.03	1.21^a ^± 0.09	0.79^b ^± 0.02	0.91^b ^± 0.02	0.88 ± 0.01	1.05a* ± 0.02
LDH	485.5 ± 14.0	1071.5^a*^ ± 5.59	891.5^a*b*^ ± 20.3	914.0^a*b*^ ± 0.01	575.5 ± 120.0	463.0 ± 30.8

Values are expressed as means ± SD; n = 10 for each treatment group.

^a ^Significant difference from the control group at p < 0.05.

^b ^Significant difference from the AlCl_3_-intoxicated group at p < 0.05.

^c ^Significant difference between AlCl_3_+FSP and AlCl_3_+FSE groups at p < 0.05.

* p < 0.001.

In brain and liver TBARS and LDH are expressed respectively as: nmol MDA/mg protein and UI/g of tissue; in plasma TBARS and LDH are expressed respectively as: nmol MDA/l and UI/l.

### Lactate dehydrogenase (LDH) activity

As shown in Table 2 AlCl_3 _treatment induced an important increase in plasmatic LDH release (+120.7%) and relatively similar increase in liver and brain (+47.43% and +36.39% respectively).

### Correlation between measured parameters

Correlation analysis of TBARS and LDH in plasma, liver and brain with plasmatic lipid and glucose profile are illustrated in Table 3.

**Table 3:** Pearson correlation coefficients assessed between parameters measured in all experimental groups.

n = 60	TBARS_Brain_	TBARS_Liver_	TBARS_Plasma_	LDH_Brain_	LDH_Liver_	LDH_Plasma_	Glc	TC	TG	HDL	LDL
**TBARS_Brain_**		0.414*	0.528**	0.509**	0.736**	0.256	0.708**	0.556**	0.490**	-0.225	0.767**
**TBARS_Liver_**			0.492**	0.480**	0.409*	0.130	0.418*	0.394*	0.280	-0.024	0.350*
**TBARS_Plasma_**				0.768**	0.613**	0.261	0.764**	0.594**	0.725**	-0.247	0.640**
**LDH_Brain_**					0.780**	0.588**	0.823**	0.783**	0.795**	-0.424**	0.821**
**LDH_Liver_**						0.559**	0.840**	0.782**	0.685**	-0.518**	0.864**
**LDH_Plasma_**							0.511**	0.530**	0.462**	-0.330*	0.583**
**Glc**								0.798**	0.769**	-0.441**	0.909**
**TC**									0.657**	-0.548**	0.669**
**TG**										-0.296	0.800**
**HDL**											-0.378*
**LDL**											

Data are presented as r values.

* Correlation is significant at the 0.05 level (2-tailed).

** Correlation is significant at the 0.01 level (2-tailed)

#### TBARS versus lipid profile

TBARS in brain, liver and plasma correlated positively with TC, TG and LDL-C but the degree of correlation is variable. Indeed, in posterior brain, TBARS was correlated mostly with LDL-C (r = 0.767; p < 0.01) whereas plasmatic TBARS correlated primarily with TG (r = 0.725; p < 0.01). In liver, correlation with all parameters was low (0.280 < r < 0.394). Regarding HDL-C, no significant correlation was recorded and it is expected because AlCl_3 _has no effect on HDL-C (Table 1).

#### LDH versus lipid profile

LDH in brain, liver and plasma correlated positively with TC, TG and LDL-C and negatively with HDL-C. Distinctions existed yet between compartments. In fact, in brain as in liver, LDH correlated strongly with TC, TG and mainly LDL-C (r = 0.821 and r = 0.864 respectively; p < 0.01). In plasma, similar mild correlations were observed for all parameters (0.462 < r < 0.583).

#### TBARS versus LDH

In brain like in liver, LDH leakage was correlated positively to TBARS in brain, liver and plasma. The most prominent correlations were recorded between plasmatic TBARS and brain LDH (r = 0.768; p < 0.01) and between TBARS in brain and hepatic LDH (r = 0.768; p < 0.01). No significant correlation was found between plasmatic LDH and TBARS in brain, liver and plasma.

#### Brain TBARS versus liver TBARS

A significant but moderate positive correlation was reported between TBARS from different compartments (r ≤ 0.528).

#### Brain LDH versus liver LDH

LDH leakage in the brain correlated positively with that observed in the liver (r = 0.780; p < 0.01). The correlation was weaker, yet statistically significant, between its levels in plasma and those in brain and liver (r = 0.588 and r = 0.559 respectively; p < 0.01).

#### Levels of glycemia versus measured parameters

Variations of all measured parameters were correlated to blood glucose level. The most prominent correlation was observed for LDL-C (r = 0.909; p < 0.01), correlations to total cholesterol and triglycerides were also high (r = 0.798 and r = 0.769 respectively; p < 0.01). Lipid peroxidation in plasma seemed more affected by glycemia than that in brain and liver (r = 0.764; r = 0.708 and r = 0.418 respectively; p < 0.01) whereas LDH release in these two organs was strongly correlated to blood glucose level (r = 0.823 and r = 0.840 respectively; p < 0.01).

#### Flavonoids and 4-Hydroxyisoleucine identification

To elucidate the structures of phenolic compounds mainly flavonoids in fenugreek seeds, the aqueous methanol extract was analyzed with diode array detection. HPLC analysis of the fractions showed the presence of peaks with flavonoid type UV spectra (two bands, λ_max _of band 1 between 320 and 350 and λ_max _of band 2 between 250 and 270 nm). Table 4 lists the identified compounds with their retention time. The structure assignment of flavonoids for which no standards were available was based on a systematic search for molecular ions using extracted ion mass chromatograms and comparing those with data in the literature. Thus, the ESI mass spectrum in positive mode of compound 2 exhibited a base peak [M + H]^+^at *m/z *449 and an aglycone ion at *m/z *287. The loss of 162 amu from the pseudo-molecular ion indicated the presence of a hexosyl moiety. The λ_max _of UV spectrum at 345 and 265 nm suggested that flavonoid 2 was a kaempferol 3-O-glucoside. Flavonoid 3 exhibited a base peak [M + H]^+^at *m/z *= 579 and also an intermediate ion at 433 and an aglycone ion at *m/z *271. The loss of 146 amu from pseudo-molecular ion indicated the loss of sugar rhamnose and the loss of 162 amu from the intermediate ion was due to the loss of glucose. The obtained MS spectra combined to the UV spectra (λ_max _at 338-266) suggested that compound 3 was apigenin 7-O-rutinoside. On the other hand, by comparing their HPLC retention time, UV spectra, and mass spectra with the data obtained from standard in-house libraries, compounds 4, 5 and 6 were identified as naringenin, quercetin and vitexin respectively. Another amino acid compound, 4-hydroxyisoleucine (1) was identified using an LC-MS apparatus in the positive ionization mode. The spectrum exhibited a molecular ion at *m/z *148 [M + H]^+ ^with fragments at *m/z *138, 130, and 118. The obtained mass fragments were consistent with those described earlier [20].

**Table 4:** Compounds detected in fenugreek seed extract with their retention times, UV spectra and mass spectral data.

N°	Flavonoid	Retention time (min)	UV λ max (nm)	[M+H]^+ ^(*m/z*)^a^	[I+H]^+ ^(*m/z*)^b^	[A+H]^+ ^(*m/z)*^c^
1	4-hydroxy-isoleucine	1.6	260	148	-	-

2	Kaempferol 3-O-glucoside	8.7	265, 345	449	-	287

3	Apigenin 7-O-rutinoside	12.3	338, 266	579	433	271

4	Naringenin	13	288, 330	273	-	-

5	Quercetin	2	371, 255	303	-	-

6	Vitexin	9.1	268, 336	433	341	-

a APCI-MS (positive mode) data for the protonated molecular ion

b APCI-MS (positive mode) data for protonated intermediate molecular ions

c APCI-MS (positive mode) data for the protonated aglycone ion

## Discussion

The major effects of aluminium-induced neurotoxicity has been related to lipid peroxidation via free radical production [21]. In the present experiment, there was a significant increase in LPO after aluminium exposure in terms of MDA levels in liver, blood and especially in brain which confirms the susceptibility of brain to oxidative insult. In brain, LPO correlated significantly with lipid profile components in plasma principally LDL-C. Thereby, it seems that the assayed endproducts of LPO in posterior brain result in a great part from LDL oxidation. Low-density lipoproteins are in fact highly vulnerable to oxidative modifications especially when it is triggered by metal ions like aluminium [22]. The strong correlation found between LDL-C and glycemia during this experiment suggest that hyperglycemia induced by aluminium ingestion is the primary cause of LDL oxidation. Glycated LDL is in fact a preferred target for oxidative modifications [23]. A similar but stronger correlation was found between LDL-C and LDH release in brain. LDH leakage has been used as a marker of Al toxicity [24]. It was found to occur simultaneously with the elevation of LPO [25] as a result of cell membrane deterioration. However, neuronal LPO is not the only cause of cerebral damage that links LDH to LDL-C. Indeed, oxidized LDL have been found to induce apoptosis of mouse cerebrovascular endothelial cells [26] which supports the hypothesis that LPO plays a role in AD through linking agents contributing to blood-brain barrier disruption [27]. On the other hand, unlike LPO, LDH leakage in posterior brain appears to be highly associated to plasmatic TC and TG levels. This might be explained in part by the link between LDH and LDL-C given that hypercholesterolemia and even hypertriglyceridemia lead to LDL-C increase and oxidation. The LDL-C/TG ratio has yet been suggested to be an important predictor of LDL oxidation [28]. Nevertheless, increasing circulating cholesterol could have a direct effect on LDH release as hypercholesterolemia enhances intra-neuronal accumulation and deposition in brain of β-amyloid protein [29], which is considered to induce oxidation [30] and plays a pivotal role in Alzheimer's disease. Changes in the extracerebral cholesterol levels could also induce modifications in brain cholesterol and low-density lipoprotein receptors present in the blood-brain barrier [31]. Otherwise, the increase in circulating cholesterol due to Al administration indicates a loss of membrane integrity [32] as it was confirmed by LDH release in brain, liver and blood. Similarly, Al exposure can result in Al accumulation in the liver leading to a disturbance of lipid metabolism and an elevation of serum cholesterol [33]. This explains the high correlation between LDH in liver and both LDH and TBARS in brain. This correlation is in fact indirect because mediated by cholesterol.

Regarding the cerebral protective effect of fenugreek seeds, we found that co-administration of fenugreek seeds either as FSP or FSE with Al reduced significantly levels of TBARS and LDH in brain. These results indicate that fenugreek seeds are endowed with antiperoxidative properties in brain which may be mediated either by direct or indirect effects on brain. It is likely that LPO and consequently LDH inhibition is owing to the antiradical and antioxidant potential of polyphenolic flavonoids of *Trigonella *seeds emphasized through *in vitro *and *in vivo *experiments [18,34-37]. Five flavonoids (kaempferol 3-O-glucoside, apigenin 7-O-rutinoside, naringenin, quercetin and vitexin) were in fact detected in this extract using LC-MS/MS. The well known hypoglycemic property of fenugreek seeds has also been used to explain its anti-peroxidative action in brain during diabetes [17,38]. However, correlations established in this study between LPO and LDH in brain and lipid profile suggest that fenugreek seeds exert their neuroprotective effect via controlling lipid and lipoprotein metabolism. Indeed, our study showed that FSP and FSE were effective in lowering plasma cholesterol, triglyceride and LDL-cholesterol in AlCl_3_-treated rats which is in line with the previous studies [20,39,40]. Several mechanisms, in addition to various components have been suggested to explain the lipid-lowering effect of fenugreek seeds. These include a direct effect on cholesterol metabolism by inhibiting the key enzymes involved in cholesterol and fatty acid synthesis. Number of studies has shown that steroid saponin extracted from fenugreek seeds has the ability to modify cholesterol status by its capacity to bind both cholesterol and bile acids [41]. Diosgenin, a steroidal sapogenin extracted from fenugreek seeds, has in fact been shown to reduce TC as well as LDL-C in high-cholesterol fed quails [42]. On the other hand, trigonelline, an alkaloid isolated from fenugreek seeds, was found able to normalize the rate of lipogenesis in streptozotocin induced hyperglycemic rats by stimulating hepatic lipogenic enzymes [43]. A recent study carried by Vijayakumar et al. [44] has proven that precipitable protein/peptide or associated factors could be responsible for improvement in serum lipid profil through hypolipidemic effect on adipocytes and liver cells leading to decreased TG and cholesterol synthesis in addition to enhanced LDL receptor-mediated LDL uptake. Nevertheless, the lipid-lowering compounds in fenugreek seeds could be of a polyphenolic nature. Indeed, Wilox et al. [45] provided evidence that naringenin not only decrease cholesterol biosynthesis but also inhibit acyl transferase (ACAT), a key enzyme involved in the esterification and absorption of cholesterol, secretion of hepatic LDL cholesterol, and cholesterol accumulation in the arterial wall. Naringenin is in fact a well known flavonoid which was detected in our extract. This effect could explain the significant increase in plasmatic HDL-C following TC decrease when fenugreek seeds are administrated to AlCl_3_-treated rats. Increasingly, hypolipidemic effect of fenugreek seeds has been attributed to the presence of 4-hydroxyisoleucine an atypical branched-chain amino acid derived from fenugreek [46] also detected in the used FSE. However, the action of 4-hydroxyisoleucine or galactomannan on lipid profile, like other components of fenugreek seeds, could be due to achievement of normoglycemia where there was no further degradation of already accumulated lipids for otherwise glucose starved cells [43]. Hypercholesterolaemia and consequently the increase of TG and LDL-C are in fact highly correlated to hyperglycemia. 4-Hydroxyisoleucine was shown to display an insulinotropic property in vitro, stimulate insulin secretion in vivo, and improve glucose tolerance in normal rats and dogs and in rat model of type 2 DM [47]. Other components of *Trigonella *seeds having hypoglycemic effects include arginine, tryptophan, ascorbic acid, niacin, nicotinic acid, chromium, copper, magnesium, manganese, zinc, gentianine, choline and quercetin, a flavonoid also detected in our extract [48]. On the other hand, the significant hepatoprotective effect of fenugreek seeds as evidenced by decreased levels of TBARS and LDH may be a secondary indirect mechanism for its neuroprotective effect, taking into account the high observed correlation between hepatic injury induced by AlCl_3 _and LPO in brain. Eventually, the decrease of LPO and LDH in brain after fenugreek administration might also be attributed to its oestrogenic constituents (saponines, trigoneosides, flavonoids) [49]. Their action could be direct since phytoestrogens have shown potential neuroprotective properties [50] or indirect thanks to their hypocholesterolemic effect [51].

Finally, it is worthwhile to mention that in this study, fenugreek seeds were given either as FSP 5% or FSE (100 mg/kg) in order to make a useful comparison as for the best form of administration. The dose of powdered fenugreek seeds was equated to the therapeutic dose suggested for humans and has been subjected to nutritional and safety evaluation [52] and the dose of fenugreek seed extract was established based on a previous study which has proved its safety and therapeutic effect [36]. Although many compounds present in the whole fenugreek seed could play a role in the described actions, similarities between the effects of the whole seed (FSP) in one hand and the effects of the seed extract (FSE) on the other hand, suggest that a mixture of flavonoids and 4-hydroxyiseuleucine was enough to generate neuroprotection against Al toxicity.

## Conclusion

Even if preliminary, the present study demonstrates that aluminium-induced LPO could arise from alteration of lipid metabolism and that is probably related to altered lipoprotein metabolism rather than a direct effect of cholesterol oxidation. Using fenugreek seeds either as FSP or FSE could be neuroprotective thanks to their antiperoxidative activity but also their ability to control hypercholesterolemia, hypertriglyceridemia and hyperglycemia. Synergism among well defined components endowed with pleiotropic actions is one of the characteristics of *Trigonella foenum-graecum *seeds. The whole fenugreek seed could be suggested as a regular nutrient to protect brain from chronic aluminium toxicity. However, further investigations are imperious to adjust quantitatively the active compounds in the fenugreek seed extract and provide a base for the development of natural drugs.

## Materials and methods

### Reagents

Aluminum chloride ((AlCl_3_, 6H_2_O), analytical grade) and all used chemicals were of analytical grade and were purchased from Sigma-Aldrich Chemical Co. (St. Louis, France).

### Animals

Sixty female Wistar rats (weighing 208-220 g) were obtained from the Central Pharmacy (SIPHAT, Tunis, Tunisia). They were fed pellet diet, purchased from the Industrial Society of Rodents' Diet (SICO, Sfax, Tunisia) and tap water *ad libitum*. Animals were kept in an air-conditioned room (temperature 22 ± 3°C and relative humidity of 40%) with a 12 h light/dark cycle. The experimental procedures were carried out according to the National Institute of Health Guidelines for Animal Care and approved by the local Ethics Committee.

### Plant material

#### Preparation of fenugreek seeds powder (FSP)

*Trigonella *seeds purchased from the local market were finely powdered and mixed at 5% in ground standard rat feed (i.e. 5 g of dry ground *Trigonella *seeds in 95 g of ground rat food).

#### Preparation of fenugreek seeds extract (FSE)

An aqueous methanol fenugreek seeds extract was prepared according to the protocol described by Kaviarasan et al. [34]. In brief, fenugreek seeds were finely powdered, mixed with 80% methanol and kept at room temperature for 5 days. The mixture was then filtered and the solvent was evaporated to get the residue. This latter was dissolved in water and the aqueous layer was washed with petroleum ether several times until a clear upper layer of petroleum ether was obtained. The lower layer was then treated with ethyl acetate containing glacial acetic acid (10 mL/L). Extraction was then carried out for 36 h at room temperature and the combined ethyl acetate layer was concentrated. The residue was lyophilised and stored at -20°C. The extract was dissolved in double-distilled water for oral administration.

#### Characterization of fenugreek seed extract by LC-MS/MS analysis

The LC-MS/MS experiments were performed as described previously by Belghith-Hadrich et al. [20] using an Agilent 1100 LC system consisting of a degasser, a binary pump, an autosampler and a column heater. The column outlet was coupled with an Agilent MSD Ion Trap XCT mass spectrometer equipped with an ESI ion source. Data acquisition and mass spectrometric evaluation were carried out in a personal computer with Data Analysis software (Chemstations). For the chromatographic separation, a Zorbax 300 Å Extend-C-18 Column (2.1 × 150 mm) was used. The flavonoids and other compounds were identified using a combination of HPLC with diode array detection and liquid chromatography coupled with an electrospry ionization mass spectrometry (ESI-LC-MS/MS) on the basis of their UV spectra and mass spectra and by comparison of the spectra with those of available authentic standards.

### Study design

Rats were treated according to the protocol established by Gong et al. [53] in which a brain dysfunction model was established. In brief, rats, randomly distributed into six groups of ten animals each, were given a daily AlCl_3 _solution (500 mg/kg, i.g, 0.5 mL/100 g) or saline (0.5 mL/100 g, i.g. for control) for the first month, and then fed with an AlCl_3 _solution (1600 ppm in drinking water) for up to 5 months. Three months after Al administration, rats were given either FSP 5% in powdered rat feed or FSE (100 mg/kg, i.g) for two months together with AlCl_3_.

### Blood and tissue collection

Blood samples were collected under anesthesia by cardiac puncture in heparinized tubes. Plasma was separated from the blood cells by centrifuging the blood at 3,000*xg *for 15 min at 4°C and stored in aliquots at -20°C until analysis. Livers and brains were removed quickly from animals, washed in ice-cold physiological saline. Then, multiple lobes of the liver from each rat were cut out, minced and homogenized (10% w/v) separately in ice-cold 1.15% KCl-0.01 mol/L sodium, potassium phosphate buffer (pH 7.4) in a Potter-Elvehjem type homogenizer. The homogenate was centrifuged at 10,000*x *g for 20 min at 4°C, and the resultant supernatant was stored at -80°C to be used for different enzyme assays.

Regarding the brain, temporal and parietal lobes were isolated from one hemisphere of each brain by free hand dissection on ice according to the method of Glowenski and Iversen [54]. Brain tissue was minced and homogenized (10% w/v) in ice-cold 0.1 M phosphate buffer (pH 7.4) then centrifuged at 10,000*x *g for 30 min at 4°C, the resultant supernatant was used for different enzyme assays.

### Biochemical assays

#### Determination of blood lipids and glucose levels

Plasmatic cholesterol, triglycerides, glucose, total protein content and HDL-C levels were quantified by enzymatic methods using commercial kits (Randox-Antrim, UK). Plasmatic LDL-C level was calculated according to the Friedewald equation [55].

#### Lipid Peroxidation Estimation

The extent of lipid peroxidation was assessed by measuring the content of thiobarbituric acid reactive substances (TBARS) following the method of Yoshioka et al. [56] in plasma and the method of Buege and Aust [57] in liver and brain. TBARS were expressed as malondialdehyde (MDA) amount using freshly diluted malondialdehyde bisdimethylacetal as standard.

#### Lactate dehydrogenase activity

The activity of LDH in plasma, liver and brain was measured using commercial reagent kits (Randox-Antrim, UK).

### Statistical Analysis

Data were expressed as mean ± standard deviation (SD) and analyzed using one-way analysis of variance (ANOVA) followed by the post hoc Tukey's test, used for comparison. Bivariate correlation between variables using Pearson's correlation coefficient r value was used. Values were considered statistically significant when p < 0.05. Statistics were done using IBM SPSS Statistics 19.

## Competing interests

The authors declare that they have no competing interests.

## Authors' contributions

YB-N conceived this study, designed it, analyzed and interpreted the data and wrote the manuscript. HB participated in the study design and data acquisition. MB carried out the plant extract characterization. IF-S carried out plasmatic lipids assays. ZH helped to draft and revise the manuscript. HBC drafted and revised the manuscript. All authors read and approved the final manuscript.
